# 

**Published:** 1871-11

**Authors:** 


### CONTENTS.

COMMUNICATIONS.

Dental Jottings.... 457
A Case, and its Perplexities.... 461
Is it a Third Tooth, or the Development of the Long Dormant Pulp of a Second Molar ?... 463

Correspondence.... 464

PROCEEDINGS OF SOCIETIES.

Annual Meeting of the Lake Erie Dental Association.... 466
Proceedings of the Kansas State Dental Society.... 484
Proceedings of the Michigan Central Dental Association.... 491
Southern Dental Association.... 497
Eastern Ohio Dental Association.... 497

EDITORIAL.

State Society.... 499
The State Board of Dental Examiners.... 499
Material Supplies for our Profession.... 500

